# A Novel Interfragmentary Technique vs. A Conventional Posterolateral Approach for Unstable Femoral Intertrochanteric Fractures in the Elderly: A Retrospective Cohort Study

**DOI:** 10.3390/medicina61040605

**Published:** 2025-03-27

**Authors:** Hakan Zora, Gökhan Bayrak, Ömer Faruk Bilgen

**Affiliations:** 1 Department of Orthopedics and Traumatology, Private Medicabil Hospital, 16140 Nilüfer, Bursa, Türkiye; mdhakanzora@gmail.com (H.Z.); ofbilgen@gmail.com (Ö.F.B.); 2 Department of the Physiotherapy and Rehabilitation, Faculty of Health Sciences, Muş Alparslan University, 49250 Merkez, Muş, Türkiye

**Keywords:** elderly, intertrochanteric fractures, hip dislocation, length of stay

## Abstract

*Background and Objectives*: Intertrochanteric fractures of the femur are common in the elderly due to the increase in longer life expectancy. However, unstable intertrochanteric fractures in the elderly population were still a significant concern for the postsurgical period after total hip arthroplasty (THA). This study aimed to compare the demographics, operative time, dislocation rate, and length of stay of the novel interfragmentary technique (IFT) and the conventional posterolateral approach (CPA) for unstable intertrochanteric femoral fractures treated with THA in the elderly. *Materials and Methods*: This retrospective study investigated community-dwelling elderly patients with type III, IV, and V unstable femoral intertrochanteric fractures according to the Evans–Jensen classification, treated with THA by a well-experienced single surgeon. The patients were separated into IFT (n = 74) and CPA (n = 67) groups. Patient demographics (age, gender, and body mass index), total surgical duration, dislocation rates, length of stay and follow-up, and complication rates were recorded. *Results*: The mean age was 80.37 years in the IFT and 80.14 in the CPA groups (*p* = 0.838). Body mass index, gender, complication, and revision rates did not differ between groups (*p* > 0.05). The mean follow-up of the IFT group was 4.15 years, and 10.25 years in the CPA group (*p* = 0.001). Total surgical duration was comparable, with 69.98 min in the IFT group and 69.55 min in the CPA group (*p* = 0.697). The dislocation rate was 2.7% (n = 2) in the IFT group and 9% (n = 6) in the CPA group (*p* = 0.109). The mean length of stay was 66.97 h in the IFT group and 67.83 h in the CPA group (*p* = 0.729). *Conclusions:* The interfragmentary surgical technique, a novel technique for unstable intertrochanteric fracture surgery, shows promising clinical outcomes. Preserving the short rotator muscles and posterior capsule utilizing the novel IFT can be advantageous for the risk of dislocation without increasing surgical duration. It can be concluded that performing THA using IFT emerges as a practical and viable procedure for treating unstable intertrochanteric fractures in elderly patients.

## 1. Introduction

Intertrochanteric fractures of the femur are common among the elderly due to the growing elderly population and longer life expectancy, accounting for about 50% of all hip fractures [1]. These fractures are typically caused by low-energy trauma, such as falls, and are known for their tendency to cause instability, which can have serious consequences for older individuals [2]. According to the Evans–Jensen classification of intertrochanteric fractures, types III, IV, and V are defined as unstable fractures [3,4]. Although studies have reported which surgical option is more appropriate in patients with unstable intertrochanteric fractures, a debate continues regarding the preferences for internal fixation and joint replacement [5]. Several reports have documented the positive clinical outcomes of surgical fixations [6,7]; however, hip replacement represents one of the core treatments for unstable intertrochanteric fractures in elderly patients [4,8,9] and may also even resolve and provide satisfactory outcomes after fixation failure [10].

Total hip arthroplasty (THA) restores the hip joint with a prosthetic implant, supports early weight-bearing activities, and helps lower the potential for complications after intertrochanteric fractures [6,11]. The functional status of patients following hip fractures are dependent on various factors, including the patient’s condition, the type of implants, and surgical approach utilized in treatment [4]. The conventional posterolateral approach (CPA) for THA has been utilized for decades after proximal femoral fractures [11,12]. However, several reports indicated higher dislocation rates after the CPA due to the cutting of the posterior capsule and external rotators in the hip joint [6,11,13]. In contrast, the prior report has demonstrated no difference in dislocation rates regardless of the approach utilized [14].

A novel surgical intervention, the interfragmentary surgical technique, has been utilized for unstable femoral intertrochanteric fractures. This technique preserves the insertion part of the short rotators and posterior capsule of the femoral head by removing the femoral head to the fracture side [15]. However, whether the interfragmentary surgical technique is superior to the conventional approach in terms of dislocation rate or vice versa needs to be clarified. Therefore, the aim of this study was to compare the demographics, operative time, dislocation rate, and length of stay of the interfragmentary surgical technique and the conventional approach for unstable intertrochanteric femoral fractures in the elderly.

## 2. Methods

### 2.1. Study Design

This retrospective study investigated community-dwelling elderly patients with type III, IV, and V unstable femoral intertrochanteric fractures according to the Evans–Jensen classification [3] in the Private Medicabil Hospital. The study was conducted in accordance with the Declaration of Helsinki, and the protocol was approved by the İstanbul Yeni Yüzyıl University Ethics Committee for Science and Health Sciences Not Requiring Medical Intervention (protocol code 2024/07-1291, approval date 8 July 2024).

### 2.2. Patients

Community-dwelling elderly patients who underwent THA due to type III, IV, and V unstable femoral intertrochanteric fractures at the Private Medicabil Hospital were investigated. A total of 138 patients were treated either by the interfragmentary technique (IFT, n = 74) with a posterolateral approach or CPA (n = 67) between January 2011 and December 2021. Inclusion criteria were the following: Evans–Jensen classification with type III, IV, and V unstable femoral intertrochanteric fractures due to low energy trauma or falls [3], being 65 years of age or older, and not having a neurological disease that causes loss of muscle strength and walking ability. The exclusion criteria were developmental hip dysplasia or pathological hip fractures, a history of high-energy trauma, previous hip surgery, and intervention on the same hip within the last three months.

### 2.3. Surgical Procedures

The THA implementation was utilized for patients who had unstable intertrochanteric fractures in our hospital. The patients in the study underwent the same type of cementless prosthesis performed by a single surgeon with 30 years of experience using the novel IFT with the posterolateral approach or CPA. Each patient had a personalized approach to trochanteric fixation, which included using a trochanteric plate and cable.

**Figure 1:**
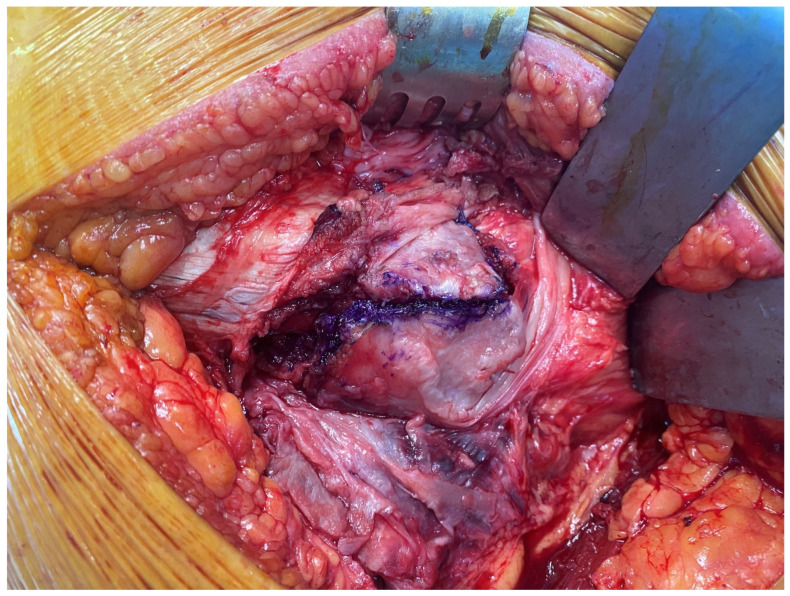
Marking of the fracture lines.

**Figure 2:**
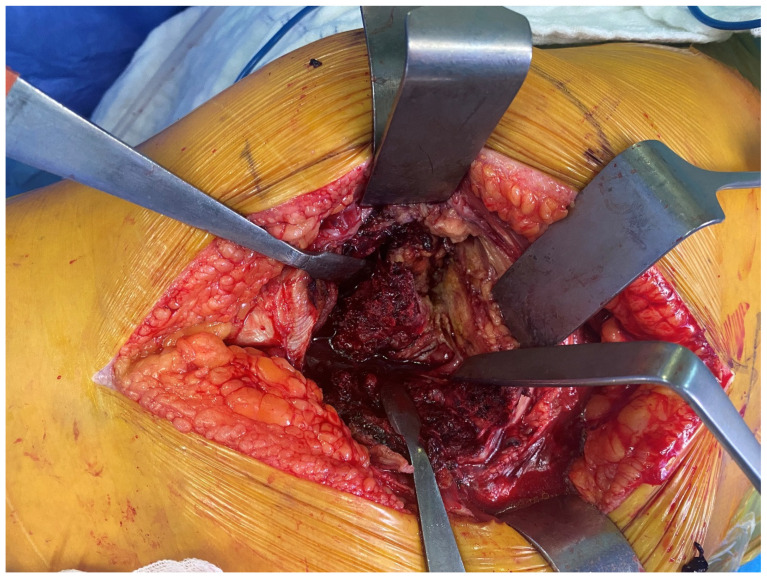
Separation of the fracture lines.

**Figure 3:**
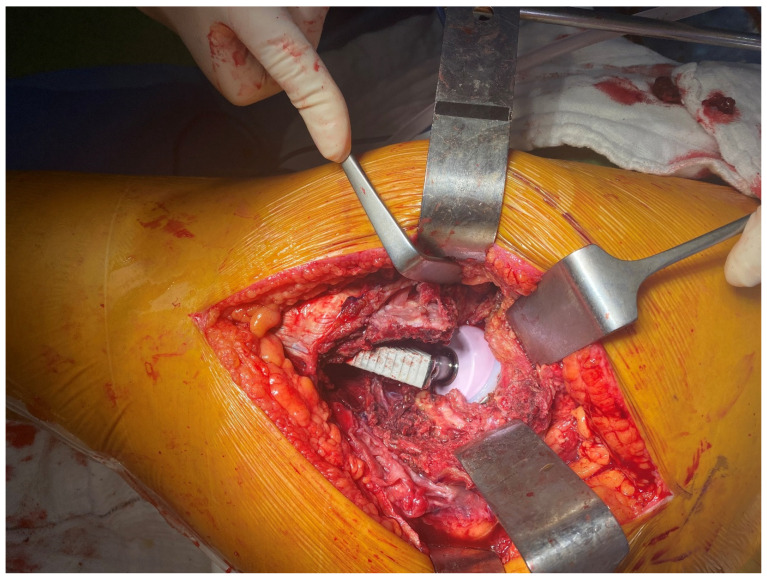
Inserting the prosthesis between the fragments.

**Figure 4:**
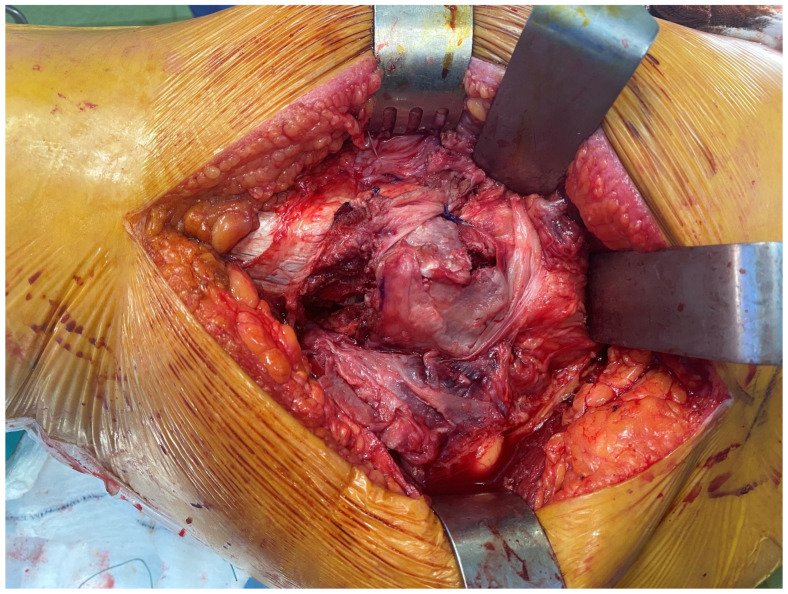
Reduction in the fragments after prosthesis placement.

*Interfragmentary technique:* The IFT was carried out using a posterolateral approach. The patient was placed on the operating table in the lateral decubitus position. The skin incision was made along the proximal posterior border of the femur. The tensor fascia latae was cut open. The bursae in the trochanteric region were excised. The gluteus maximus muscle fibers were opened proximally. The fracture fragments of the trochanter major were identified (Figure 1). Then, the femoral neck and head were reached by separating the muscle tissues parallel to their fibers and entering through the fractured fragments. The fractured femoral head and neck were removed (Figure 2). The piriformis and other short external rotators, and posterior capsule were preserved. After labrum excision, the acetabular component was placed in the appropriate position. Then, the femur was placed in an internal rotation position, and the femoral stem was placed in the proper position and height (Figure 3). Reduction and trochanteric plate were placed for trochanteric fracture, and fixation with a cable was provided (Figure 4).

*Conventional posterolateral approach:* The patient was positioned on the operating table in the lateral decubitus position. A skin incision was made along the proximal posterior border of the femur. The surgeon cut open the tensor fascia latae and excised the bursae in the trochanteric region. The gluteus maximus muscle fibers were then opened proximally. The piriformis and other short external rotators were detached from their attachment to the trochanter and overturned over the sciatic nerve. A “T” shaped incision was made in the joint capsule to reach the femoral head. The fractured femoral head and neck were removed, and after excising the labrum, the acetabular component was placed in the appropriate position. The femur was then placed in an internally rotated position, and the femoral stem was positioned at the proper height. A trochanteric plate was used for a trochanteric fracture, and fixation with a cable was provided. Finally, the capsule, piriformis, and short rotators were sutured back into their original positions.

### 2.4. Physical Therapy

All patients in our study received comprehensive education on prosthesis protection and dislocation before and after THA surgery. The patients received standardized physical therapy regimen initiated at the postoperative first day after the THA implementation. The physical therapy regimen included the ankle pump, cold pack, isometric quadriceps setting, transfer activities, active-assistive hip/knee range of motion exercises, and partial weight-bearing activity using walker during hospitalization. After discharge, patients received home-based exercise protocol, including partial weight-bearing activities, straight leg raise, quadriceps strengthening, and massage near to the surgical incision. The stitches were removed on days 15–19 and the patients started outpatient physical therapy for 5 days a week for 4 weeks at our hospital. The participants were recruited using a one-by-one approach by specialized physiotherapists for the standardized physical therapy regimen at the hospital. The outpatient physical therapy regimen comprised of isotonic quadriceps strengthening, hip abduction, hip flexion and hip extension with elastic band resistance, chair stands, side walking, stepping up and down, and rising on tiptoes exercises. After the outpatient physiotherapy regimens were completed, the patients were instructed to walk from 20 to 30 min daily.

### 2.5. Outcomes

The study involved a retrospective investigation of the medical records of all patients. We collected data on the patient demographics (age, gender, and body mass index). Operative data, including the surgical technique type, and the total surgery duration were recorded. Mean follow-up and length of stay, dislocation rate, superficial infection and nonunion complications, and revision rates were also collected.

### 2.6. Statistical Analysis

Data obtained in the study were analyzed statistically using SPSS 27.0 software (IBM Corp., Armonk, NY, USA). The conformity of the data to normal distribution was examined with the Shapiro–Wilk test. Descriptive statistics were stated as mean ± standard deviation (95% confidence interval) values for continuous variables and as number (n) and percentage (%) for categorical variables. A Student *t*-test was used to explore the means between the two groups. The Pearson Chi-square test was used for categorical data analyses. The statistical significance level was set as *p* < 0.05.

## 3. Results

A total of 141 patients’ data were analyzed in the study. Notably, the mean age was almost identical at 80.37 years in the IFT and 80.14 in the CPA groups (*p* = 0.838). Similarly, the body mass index was almost equal at 25.76 kg/m^2^ in the IFT and 25.74 kg/m^2^ in the CPA groups (*p* = 0.955). The IFT group was comprised of 27 males (36.5%) and 47 females (63.5%), and the CPA group included 19 males (28.4%) and 48 females (71.6%) (*p* = 0.304). Right dominance was present 94.6% (n = 70) in the IFT and 95.5% (n = 64) in the CPA groups (*p* = 0.800). The IFT group consisted of 25 dominant extremity-affected patients (33.8%), whereas the CPA group was 39 (58.2%) (*p* = 0.004). Patients’ descriptive data are shown in Table 1.

A comparison of follow-up, surgical duration, length of stay, and dislocation and complication rates of the groups are presented in Table 2. The mean follow-up of the IFT group was 4.15 years, whereas the CPA group was 10.25 years (*p* = 0.001). Total surgical duration was comparable, with 69.98 min in the IFT group and 69.55 min in the CPA group (*p* = 0.697). The mean length of stay was 66.97 h in the IFT group, and 67.83 h in the CPA group (*p* = 0.729). The dislocation rate was 2.7% (n = 2) in the IFT group, and 9% (n = 6) in the CPA group (*p* = 0.109). Trochanteric nonunion was present in 1.4% (n = 1) in the IFT group and none in the CPA group (*p* = 0.340). The IFT group had a periprosthetic fracture (n = 1, 1.4%), and both the IFT and CPA groups had superficial infections (n = 1, 1.4%) (*p* = 0.633). There were no perioperative complications in any of the patient groups.

## 4. Discussion

Falling-related fractures are growing due to higher life expectations in the elderly [16]. Intertrochanteric fractures are particularly prevalent among the elderly population, have a significant impact on their overall health, and could increase mortality and morbidity rates [4,7]. The primary objective of surgery for elderly patients with unstable intertrochanteric fractures is to achieve pain relief, facilitate early mobilization and function, and support rehabilitation [4].

Prior research examined the THA via posterolateral approach after unstable intertrochanteric fractures, and the main respect of the outcomes was the prosthesis’s dislocation [13,14,17]. While prosthetic dislocation is not a common occurrence, it represents a significant concern for patients due to the need for revision surgery and causes dissatisfaction [13,14]. Surgical techniques have typical complications of variable frequency, and the overall dislocation rate for hip arthroplasty is approximately 4–8% [18,19,20]. Earlier research on unstable intertrochanteric fractures after THA using a posterolateral approach in the elderly population showed dislocation rates of 1.58% [21], 1.9% [18], and 3.77% [8]. Our study showed that the novel interfragmentary surgical technique with the posterolateral approach results in only 2.7% of dislocations for an average of 4.15 years follow-up compared to the conventional approach (9%). Our novel interfragmentary surgical technique, which preserves the piriformis and other short rotators and posterior capsule, proposes promising dislocation rates from a clinical point of view compared to the conventional method and prior published reports.

The duration of the surgical operation is affected by various parameters, such as surgeons’ technical performance and overall experience, surgical technique, and complications [18,22]. Some studies reported that surgical duration using the posterolateral approach were 66.6 min [12], 73.6 min [11], 77.5 min [23], and 80.25 min [2] in hemiarthroplasty surgery and 72 min [15], 78.08 min [6], and 110 min [8] in THA surgery in elderly patients. Only one previous retrospective study, carried out in our hospital, used the IFT and reported a 68 min surgical duration in unstable intertrochanteric fractures [15]. Our findings in both groups revealed a mean surgical duration of 69.98 min for the IFT and 69.55 min for the CPA. Considering the earlier findings, this study’s outcomes support the notion that the novel interfragmentary surgical technique and CPA can achieve shorter surgical durations for unstable intertrochanteric fractures than previous studies on hemiarthroplasty and THA, with the advantages of an experienced surgeon and lower perioperative complication.

THA uses artificial femoral and acetabular prostheses, but pre-and postoperative conditions can delay recovery and lead to longer hospital stays [6,24]. Prior research investigated the length of stay after unstable intertrochanteric fracture surgery, which lasted for 6.55 days [2], 6.9 days [23], and 16.63 days [25] in hemiarthroplasty patients, and 9.5 days [8] and 7.67 days [6] in THA patients. Additionally, the length of hospital stay was significantly associated with the postsurgical complications [24]. Elderly patients undergoing surgery for unstable intertrochanteric fractures face numerous postoperative complications, with periprosthetic fractures being a major concern following THA [26]. Initiating early mobilization and weight-bearing postsurgery is crucial in reducing the risk of complications linked to prolonged immobilization [16]. However, in the current study, patients undergoing both surgical procedures had almost equal hospitalization time for the length of stay: 66.97 h in the IFT and 67.83 h in the CPA, which indicates nearly three days. One patient (1.4%) in the IFT had periprosthetic fracture complication, and a superficial infection was noticed in one patient (1.4%) in each group. An early physiotherapy regimen provided for all patients in this study, along with preoperative and postoperative education on prosthesis protection, may have contributed to lower hospitalization time and the prevention of other potential complications in elderly patients.

The main strength of this study is its revelation of the impacts of the novel interfragmentary surgical technique on unstable intertrochanteric fractures in the elderly population. These include showing that the risk of dislocation can be reduced from a clinical perspective by preserving the piriformis muscle and posterior capsule during the posterolateral lateral approach in unstable intertrochanteric fractures. In addition, this novel technique does not prolong the surgical time compared to the conventional method. Since the piriformis muscle, along with the other short external rotators and the posterior capsule, remains intact in our novel interfragmentary surgical approach, the risk of damage to the sciatic nerve is considerably reduced. Accordingly, we posit that the likelihood of complication of the low foot will also be diminished, representing a substantial advantage of this technique. This study has limitations. First, we did not have the mental health status of the patients, which could have affected dislocation rates and hospital stays. Second, we do not have the mortality rates of the patients after interfragmentary and conventional posterolateral surgeries.

## 5. Conclusions

The interfragmentary surgical technique, a novel approach for managing unstable intertrochanteric fractures, demonstrates promising clinical outcomes. By preserving the short rotator muscles and the posterior capsule, this technique offers a potential advantage in reducing the risk of postoperative dislocation from a clinical perspective without increased surgical duration, a critical concern in the surgical treatment of such fractures. Given these findings, THA performed using the interfragmentary surgical technique emerges as a clinically viable and practical intervention for managing unstable intertrochanteric fractures, particularly in elderly patients, where the dislocation risks are of great importance. Further prospective randomized-controlled studies, including larger samples investigating the interfragmentary surgical technique’s long-term outcomes, are needed.

## Figures and Tables

**Table 1:** Demographic data comparisons of the groups.

Variables	Interfragmentary Technique (n = 74)		Conventional Posterolateral Approach (n = 67)			
	Mean ± SD	95% CI	Mean ± SD	95% CI	t	*p*
**Age (year)**	80.37 ± 7.14	78.73–82.03	80.14 ± 6.07	78.67–81.63	0.204	0.838
**Body mass index (kg/m^2^)**	25.76 ± 2.13	25.21–26.31	25.74 ± 2.34	25.17–26.31	0.057	0.955
	**n**	**%**	**n**	**%**	**χ^2^**	***p***
**Gender**						
Female	47	63.5	48	71.6	1.057	0.304
Male	27	36.5	19	28.4		
**Dominant Extremity**						
Right	70	94.6	64	95.5	0.064	0.800
Left	4	5.4	3	4.5		
**Affected Extremity**						
Dominant	25	33.8	39	58.2	8.463	**0.004**
Nondominant	49	66.2	28	41.8		

SD, standard deviation; 95% CI, 95% confidence interval for means; kg, kilogram; m, meter; t, independent samples *t*-test; χ^2^, Pearson Chi-square statistics. Significant *p* values are presented in bold.

**Table 2:** Functional clinical data comparison between the groups.

Variables	Interfragmentary Technique (n = 74)		Conventional Posterolateral Approach (n = 67)			
	Mean ± SD	95% CI	Mean ± SD	95% CI	t	*p*
**Mean follow-up (years)**	4.15 ± 1.73	3.75–4.55	10.25 ± 2.03	9.76–10.76	−18.601	**0.001**
**Surgical duration (minutes)**	69.98 ± 6.94	68.38–71.59	69.55 ± 6.36	67.98–71.11	0.390	0.697
**Length of stay (hours)**	66.97 ± 11.53	64.30–69.64	67.83 ± 12.43	64.78–70.89	−0.347	0.729
	**n**	**%**	**n**	**%**	**χ^2^**	***p***
**Dislocation**						
Yes	2	2.7	6	9	2.569	0.109
No	72	97.3	61	91		
**Trochanteric nonunion**						
Yes	1	1.4	0	0	0.912	0.340
No	73	98.6	67	100		
**Cause of revision other than dislocation**						
Infection	1	1.4	1	1.5	0.916	0.633
Periprosthetic fracture	1	1.4	0	0		
None	72	97.2	66	98.5		

SD, standard deviation; 95% CI, 95% confidence interval for means; kg, kilogram; m, meter; t, independent samples *t*-test. χ^2^, Pearson Chi-square statistics. Significant *p* values are presented in bold.

## Data Availability

Due to privacy and ethical restrictions, the data are not publicly available.
